# Integrated transcriptomic profiling reveals immune and molecular stage-specific signatures of colorectal cancer in hispanics living in Puerto Rico

**DOI:** 10.3389/fimmu.2026.1838210

**Published:** 2026-08-11

**Authors:** Camille N. Zenón-Meléndez, Sheila N. López-Acevedo, Josué Pérez-Santiago, Yakshi N. Ortíz-Maldonado, Hilmaris Centeno-Girona, Elba V. Caraballo-Rivera

**Affiliations:** 1 Division of Shared Resources and Scientific Operations, University of Puerto Rico Comprehensive Cancer Center, San Juan, Puerto Rico; 2 Division of Clinical and Translational Cancer Research, University of Puerto Rico Comprehensive Cancer Center, San Juan, Puerto Rico

**Keywords:** colorectal cancer, cytokines, hispanics living in Puerto Rico, RNA-Seq, stage-specific, Wnt signaling

## Abstract

**Introduction:**

Colorectal cancer (CRC) remains one of the most diagnosed malignancies worldwide and continues to rank among the leading causes of cancer-related death. Cancer progression involves dynamic transcriptional and immunological changes, and although many key molecular alterations driving CRC progression have been described, stage-specific differences remain poorly understood. This study aimed to characterize transcriptomic changes in Hispanics living in Puerto Rico (PRH) to identify relevant pathways, target genes, and stage-specific immune and molecular signatures.

**Methods:**

We conducted RNA sequencing (RNA-seq) from colorectal tissues, including early-stage CRC (stages I & II), advanced-stage CRC (stage III only), and adjacent mucosal tissue, to elucidate key mechanisms that modulate CRC progression.

**Results:**

Transcriptomic analysis revealed distinct gene expression patterns across stages, with a total of 1666 and 1955 differentially expressed genes at early and advanced stages, respectively. Early-stage CRC was significantly enriched for biological pathways associated with immune regulation, including cytokine signaling, epithelial barrier function, and tissue repair mechanisms, reflected in part by activation of components of the Wnt signaling pathway. In contrast, advanced-stage (stage III) CRC displayed immunosuppressive transcriptional profiles and activation of tumor-promoting pathways, including TNF signaling, enhancement of IL-17 and Wnt signaling pathways, NF-κB signaling, angiogenesis, epithelial-mesenchymal transition (EMT), and stromal remodeling.

**Discussion:**

Pathway enrichment analyses supported a shift from immune engagement and matrix remodeling in early CRC to immune evasion and pro-metastatic programs in advanced-stage disease (comprised of stage III; metastatic stage IV was not sampled). While Wnt signaling components were differentially expressed across stages, they appeared as part of a broader network of developmental and inflammatory signals driving progression. This study provides preliminary transcriptomic evidence to inform stage-specific biomarker discovery in PRH CRC that warrants further validation in larger cohorts.

## 1 Introduction

Colorectal cancer (CRC) is the third most commonly diagnosed cancer and the second leading cause of cancer-related mortality worldwide (1, 2). In the United States (US), CRC accounts for approximately 152,810 new cases and 53,010 deaths annually (2). In Puerto Rico, it represents 11% of all cancer diagnoses and 13% of cancer-related deaths (3). Since the 1990s, CRC incidence and mortality have declined mainly due to the implementation of screening programs, the removal of precancerous polyps, and advances in treatment (4, 5). However, the rate of these improvements has slowed in recent years (2). CRC is typically categorized in four stages according to clinical progression: stages I & II (Early-stage CRC) and stages III & IV (Advanced-stage CRC), often diagnosed at advanced stages when treatment options are limited (6). Challenges to early detection and cancer control persist, including a rising incidence among younger adults and differences in outcomes across patient populations (4, 5, 7). Together, these trends highlight the need to investigate molecular and biological pathways that could support the development of more effective strategies for early disease detection.

CRC develops through a multistep process driven by the accumulation of genetic, epigenetic, environmental, and molecular factors that contribute to disease progression (8–12). Importantly, CRC should be understood not solely as a disease of structural molecular alterations, but as one in which structural and immunological mechanisms operate in concert, and whose interaction remains poorly defined, particularly in genetically distinct populations such as Hispanics (13–15). Classical models have shown that adenoma-to-carcinoma progression involves chromosomal instability, microsatellite instability, and mutations in key oncogenes and tumor suppressors, including KRAS, and BRAF (16, 17). Among these molecular alterations, dysregulation of developmental signaling pathways has emerged as a central driver of colorectal tumor initiation and progression. At a molecular level, the Wnt/β-catenin pathway is the most frequently altered signaling cascade in CRC, with disruptions observed in more than 90% of cases due to *APC* or *CTNNB1* mutations (18). Alterations in this pathway prevents β-catenin degradation, leading to its nuclear translocation and the subsequent upregulation of oncogenic targets such as *MYC*, *CCND1*, and matrix metalloproteinases (MMPs) contributing to unchecked cellular proliferation and tumorigenesis (18, 19). Beyond Wnt signaling, other pathways including TGF-β, Notch, and PI3K/Akt, play important roles in regulating cell proliferation, differentiation, and survival while maintaining tissue homeostasis, and crosstalk among these pathways further amplifies tumorigenic signaling (18, 20). Furthermore, the interaction between these oncogenic signaling and regulatory networks influences the tumor microenvironment (TME), which plays a central role in modulating CRC behavior and therapeutic resistance.

CRC can reflect a pro-inflammatory state that changes along with CRC progression (12). Pro-inflammatory cytokines can modulate Wnt signaling and vice versa, suggesting a complex co-regulation that enhances tumor growth and immune evasion. This structural-immunological crosstalk may be further shaped by population-specific genetic variation, highlighting the importance of studying CRC in diverse cohorts (14, 21). Functional studies have identified specific cytokines as key regulators of this process. For instance, IL-17 promotes inflammation and contributes to immune surveillance escape (22), while IL-4 and IL-8 are mainly upregulated at early stages of CRC (19, 23–25).

To address the molecular heterogeneity of CRC, transcriptomic studies have emerged as powerful approaches for the quantitative study of gene expression. Advances in transcriptomic technologies now enable the measurement of cellular transcripts, including messenger RNA, microRNA, and other non-coding RNAs (26–28). These approaches are broadly used in cancer research to uncover RNA-level alterations, define gene expression patterns, and identify novel targets (28, 29). Moreover, gene expression at a transcription level provides information on gene dynamics, regulation, and biological processes that provide guidance on network regulation, revealing biomarker candidates with potential diagnostic and therapeutic relevance (28–30). However, despite extensive efforts to identify biomarkers for colorectal cancer, gene expression and regulation mechanisms vary across populations (31–34). This variability shows the importance of transcriptomics profiling to understand CRC-driven signaling, inflammatory networks, and its diversity across populations. However, data on the transcriptomic landscape of CRC in Hispanic living in Puerto Rico (PRH) remains limited, even though this population shows one of the highest age-standardized CRC incidence rates among US groups (7).

Taken together, the structural and immunological complexity of CRC, compounded by the genetically distinct and underrepresented nature of PRH population (a group with a distinct genetic admixture of European, African, and Amerindian ancestry) underscores the urgent need for population-specific molecular characterization (15, 35). This study addresses that gap by providing stage-stratified transcriptomic profiling dedicated to assessing gene expression, network dysregulation, and immune-related pathway alterations in PRH CRC. In doing so, we identified potential stage-specific biomarkers candidates and pathway alterations grounded in the unique biology of this population that may inform future early detection strategies.

## 2 Materials and methods

### 2.1 Study design and sample preprocessing

**Figure 1:**
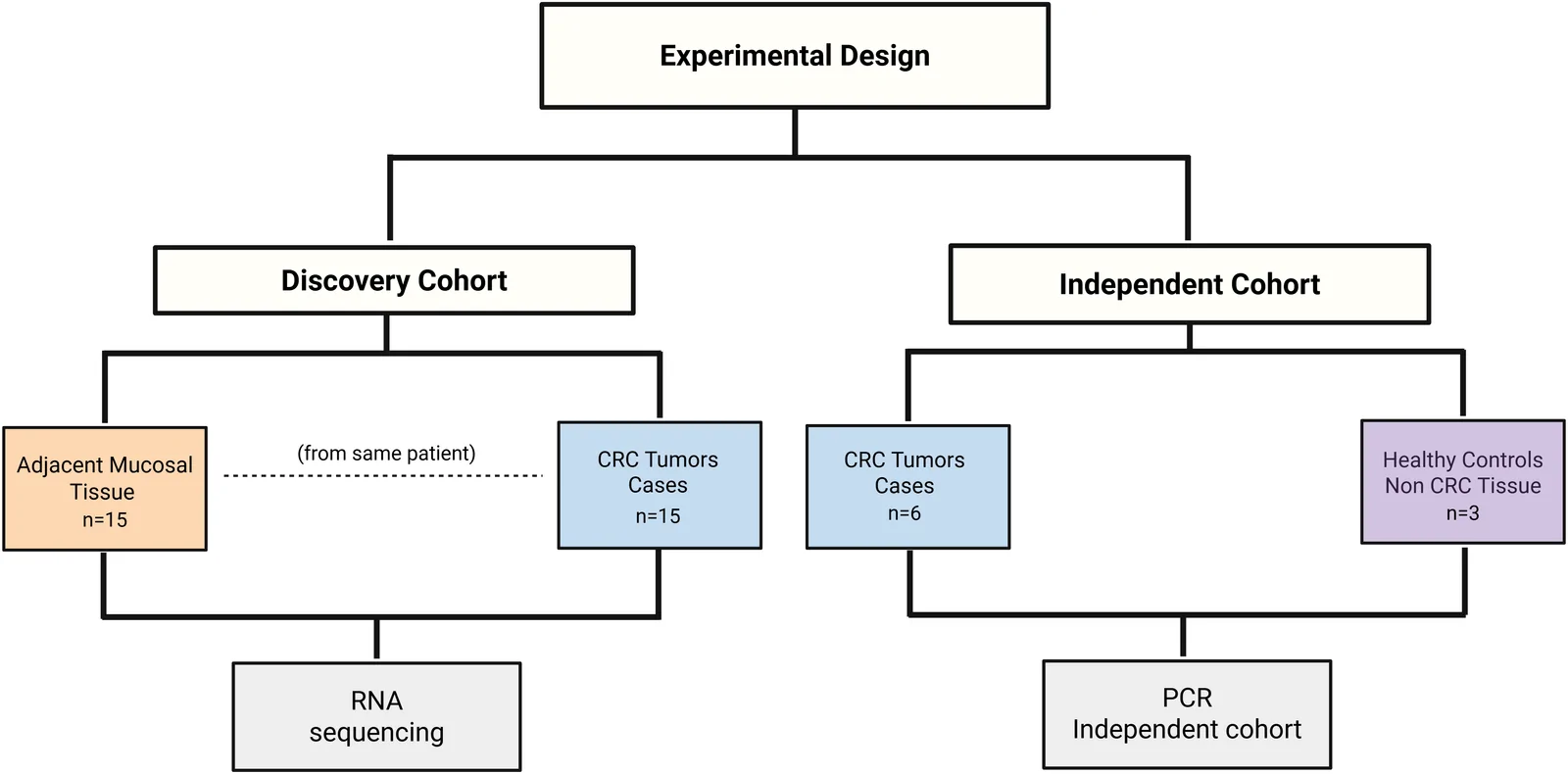
Experimental design and sample stratification. Schematic overview of the study design showing the cohort groups analyzed. Discovery cohort consists of CRC tumor tissues (n=15), stratified into early-stage CRC (n=7) and advanced-stage CRC (n=8) and adjacent mucosal tissue within the same patients (n=15). Discovery cohort was selected for RNA sequencing analysis. Independent cohort includes CRC tumor tissues (n=6), stratified into early-stage CRC (n=3) and advanced-stage CRC (n=3) and healthy control (non-CRC) tissues (n=3). Independent cohort was selected for PCR assays. Created in BioRender. Zenón, C. (2026) https://BioRender.com/ccn8b7d. Publication licensed under CC BY 4.0.

This study uses previously collected tissue from PRH enrolled in the Puerto Rico Familial Colorectal Cancer Registry (PURIFICAR) IRB #2290034759. Eligible participants were adults (≥21 years) with pathology-confirmed CRC without hereditary cancer syndromes or inflammatory bowel disease. Control participants were confirmed to have a negative colonoscopy with no colorectal adenomas. Tissue samples were collected between 2011 and 2019 for the discovery cohort and between 2008 and 2020 for the independent cohort. Samples were snap-frozen immediately upon collection and stored at −150 °C using standard protocols (36); freeze-thaw cycles were strictly avoided prior to analysis. No direct contact with human subjects was required. The experimental design protocol is illustrated in **Figure 1**. All procedures and research protocols from this secondary data analysis were approved by the University of Puerto Rico, Medical Sciences Campus Institutional Review Board (IRB) (Protocols #2290033741 & #2410296903).

Two sets of participants were selected for (1) gene discovery through RNA-seq analyses and (2) independent cohort expression profiling analysis by PCR. The discovery cohort consisted of 15 participants with early or advanced-stage CRC (stage III). Tumor and adjacent mucosal tissue were collected from each participant. Sociodemographic and clinicopathological characteristics from each participant in the discovery cohort were summarized in **Supplementary Table 1**. The independent cohort consisted of 9 participants, including tissues from healthy participants as well as tumor tissue from participants with early or advanced-stage CRC. The Kruskal-Wallis rank sum test and Fisher’s exact test were used to compare clinicopathological characteristics between groups for the independent cohort (**Supplementary Table 2**). All statistical analyses were performed using R, version 4.4.1 (R Foundation for Statistical Computing) and R-Studio Integrated Development Environment for R (Posit Software, PBC). Significance was set at 0.05.

### 2.2 RNA sequencing and bioinformatic analyses

Total RNA was extracted from 15 CRC (8 Advanced and 7 Early-Stage) tumors and adjacent mucosal tissue (30 mg) using the RNeasy Kit (QIAGEN, Maryland, USA), following the manufacturer’s protocol. RNA concentration and integrity were assessed using a NanoDrop 2000c spectrophotometer (Thermo Fisher Scientific, MA, USA; software version 1.6.198) and Agilent Bioanalyzer, respectively. Only samples with A_260_/A_280_ ratios between 1.8 and 2.0 and RNA integrity number (RIN) values exhibiting a RIN > 7 were selected for library construction (37). RNA messenger libraries were prepared using the Illumina Stranded Total RNA Prep Kit according to the manufacturer’s protocol. Paired-end sequencing (2 x 75bp) was performed on the Illumina NextSeq 550 platform following the manufacturer’s protocol.

Quality assessment of the raw FASTQ reads was conducted using FastQC (38). Adapter trimming and low-quality base removal were performed using Trimmomatic (39), followed by alignment of the reads to the human reference genome GRCh38 (hg38) using STAR (40). Gene-level quantification was carried out using RSEM (41), and the resulting expression data was imported into R/RStudio (42, 43) using tximport R package. A Principal Component Analysis (PCA) was generated to assess sample variability and clustering based on gene expression levels. Differential Gene Expression Analysis (DGEA) was performed with the DESeq2 package (v1.42.1) to compare transcriptomic profiles between CRC tumors and adjacent mucosal tissue. These analyses were conducted separately for early and advanced CRC stages. Genes were considered differentially expressed if the absolute log2 fold changes (LFC) > 2 and the adjusted p-value < 0.05. Differential gene expressions were assessed using Venn diagrams and volcano plots. Protein-protein interaction networks were inferred with differentially expressed genes (DEGs) using STRING-db (v12.0) with a score threshold of 900 and visualized with igraph (v2.1.4). Networks with 5 or more vertices were selected. KEGG-based Gene Set Enrichment Analysis was performed on the complete, log2 fold change-ranked gene list using clusterProfiler (v4.10.1). KEGG pathways were classified as upregulated or downregulated based on the normalized enrichment score (NES), with NES > 0 indicating upregulation and NES < 0 indicating downregulation.

### 2.3 Independent expression profiling of candidate Wnt targets by quantinova LNA PCR assays

To provide a complementary perspective to our initial findings, a subset of DEGs was selected for independent expression profiling (n=9) through quantitative polymerase chain reaction (qPCR) based on statistical significance, fold change, and biological relevance. Due to limited availability of CRC tumors and adjacent mucosal tissue following RNA library preparation, an independent profiling assay was performed using CRC tumors (early n=3 and advanced n=3) and healthy control tissues (n=3) obtained from independent patients, rather than matched adjacent mucosa from the discovery cohort. This approach is consistent with published evidence demonstrating molecular crosstalk between tumor and adjacent mucosa in CRC, supporting the use of non-adjacent healthy tissue as an independent expression baseline when paired samples are unavailable (44).

Expression of Wnt signaling components was profiled using the QuantiNova LNA PCR Assay Human Wnt Signaling Pathway Panel (QIAGEN, GeneGlobe ID: SBHS-043Z) and the QuantiNova LNA PCR Assay Human Wnt Signaling Targets (QIAGEN, GeneGlobe ID: SBHS-243Z). One-step PCR amplification was performed using the QuantiNova SYBR Green RT-PCR Master Mix (QIAGEN) according to the manufacturer’s protocol. Each 20 µl reaction included ROX as a reference dye and was performed using a QuantStudio 1 System (Thermo Fisher Scientific) under the cycling conditions according to the manufacturer’s protocol. Gene expression levels were normalized to the mean fluorescence of GAPDH internal reference gene. A consistent threshold above background noise for Ct values or fluorescence intensity was determined across all samples. Target gene expression levels were quantified relative to GAPDH endogenous control. The data was stratified and analyzed by the CRC stage. A heatmap was generated using GraphPad Software v.10.4.1 (Boston, USA). Statistical analysis for qPCR expression profiling was performed using one-way ANOVA, followed by Tukey’s multiple comparisons test in GraphPad Prism (v.10.4.1).

## 3 Results

### 3.1 Demographic and clinicopathological characteristics of study participants

The discovery cohort included 15 CRC participants, 8 (53.3%) with advanced-stage and 7 (46.7%) with early-stage CRC (**Supplementary Table 1**). The participants’ median age at recruitment was 63.5 years (41.1-84.5), and the median age at diagnosis was 61.0 years (41.0-84.0). Sixty percent of participants were male, and 60% were married. Participants’ education level was heterogeneous: 60% held a bachelor’s degree or higher, and the remainder reported an associate degree or lower. The cohort’s median body mass index (BMI) was 25.8 kg/m^2^ (17.5-34.0), and 28.6% of participants had diabetes. Thirty percent of participants used statins, 14.3% used aspirin, and 14.3% used NSAIDs as regular medication. Lifestyle factors showed that 35.7% of participants were lifetime smokers, and 61.5% reported lifetime alcohol consumption. Interestingly, a family history of cancer was reported by 57.1% of the participants, while family history of colorectal cancer or Lynch syndrome-associated cancers was rare (7.1% each). All 15 participants underwent colorectal surgery, with a median age at surgery of 63.0 years (41.0-84.0). Tumor sites were reported by primary locations as follows: colon 66.7% left, 13.3% right, 13.3% unspecified, and 6.7% Rectum. Pathological staging revealed a distribution of Stage I (20.0%), Stage II (26.6%), and Stage III (53.4%). Further tumor classification, regarding T and N status for each sample, is described in **Supplementary Table 1**.

The independent cohort consisted of 9 participants who were also stratified into early-stage CRC, advanced-stage CRC, and healthy control group, with each group consisting of 3 (33.3%) participants. Sociodemographic and clinicopathological characteristics remained largely consistent across these groups, showing no statistically significant differences. The median age at tissue sampling was relatively consistent, ranging from 57.0 years in the control group to 66.0 years in the early-stage group (p = 0.4). Physiological metrics such as height and weight were comparable across the cohort. Tumor location and pathological staging were recorded for all subjects. Early-stage tumors (Stage I: 33.3% and Stage II: 66.7%) were primarily located in the colon (100%). Conversely, advanced-stage tumors (Stage III: 100%) were distributed as follows: colon 33.3% left, 33.3% unspecified, and 33.3% rectum across the rectosigmoid colon, rectosigmoid junction, and transverse colon. Additional details related to the independent cohort are included in **Supplementary Table 2**.

### 3.2 Transcriptomic profiling identifies stage-specific differentially expressed genes between CRC and adjacent mucosal tissue

**Figure 2:**
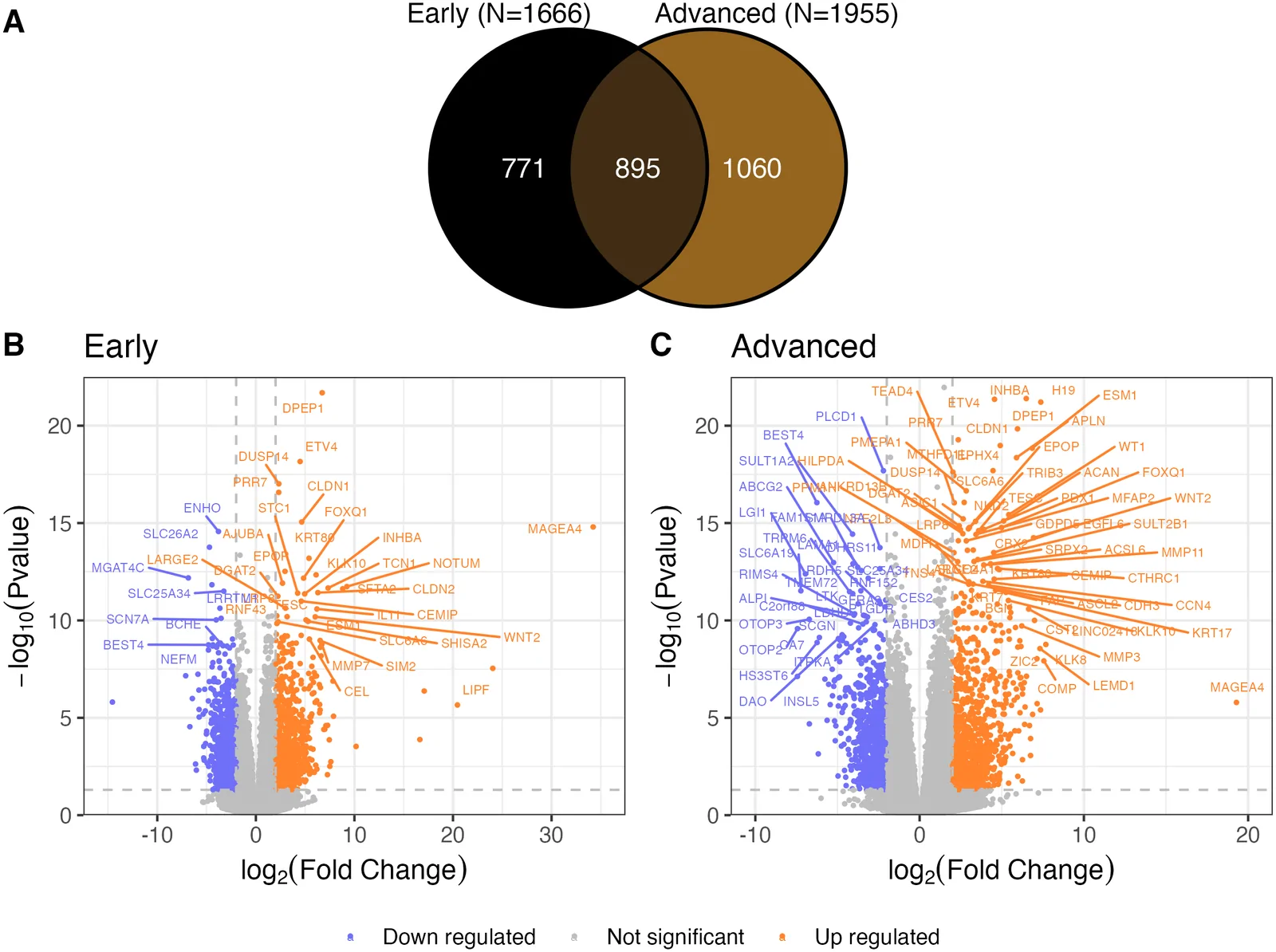
Differential gene analysis of colorectal cancer on early and advanced stages. DEGs from RNA-seq analysis of colorectal cancer samples were represented using a Venn Diagram and Volcano Plots. **(A)** Venn diagram based on DEGs between early- stage CRC and advanced-stage CRC compared with adjacent mucosal tissue. Diagram shows the shared and overall and unique number of upregulated and downregulated DEGs identified. **(B)** Volcano plot representing the DEGs of early-stage CRC and **(C)** advanced stage CRC. DEGs’ significance was based on satisfying the criteria of log 2 (fold-change) value >2 or <-2 and P<0.05. Significantly upregulated genes are represented as orange dots and downregulated genes as blue dots.

PCA of the discovery cohort transcriptome confirmed robust separation between tumor and adjacent mucosal tissue, indicating that biological signal predominates over technical noise and that no major batch effects were detected (PC1: 11.14%); **Supplementary Figure 1A**). Upon stratification by CRC stage, this tissue-based separation was preserved within each stage, further supporting the integrity of sample collection and the suitability of the dataset for downstream differential expression analyses (**Supplementary Figures 1B, C**). A total of 2726 genes were DEGs between tumors and adjacent mucosal tissues in both CRC stages (**Figure 2A**). Among the 2726 DEGs, 771 were unique to early and 1060 advanced-stage CRC, while 895 were shared among both CRC stages. **Figures 2B, C** show volcano plots that highlight the most significant DEGs per CRC stage that included *WNT2*, *WNT5A*, *WIF1*, and *IL-17A* genes in both CRC stages. **Supplementary Figure 2** highlights the top 10 significantly upregulated and downregulated DEGs in early and advanced stages, based on the significance criteria (log_2_ FC [LFC] and p-value). Among the top significantly DEGs in early-stage CRC were: *MAGEA4* (LFC 34.22), *LIPF* (LFC 20.45), *MUCL1* (LFC 10.16), *NOTUM* (LFC 9.23), *OTOP2* (LFC -5.76), *CA1* (LFC -5.78), and *MGAT4C* (LFC -5.84). *MAGEA4* (LFC 19.29) remained the top upregulated gene in advanced-stage CRC. Among the top DEGs in advanced-stage CRC were: *KLK8* (LFC 7.69), *H19* (LFC 7.37), *KLK7* (LFC 7.36), *WNT2* (LFC 6.94), *CA7* (LFC -6.1), *OTOP3* (LFC -6.72), *OTOP2* (LFC -7.43), and *INSL5* (LFC -7.44). A complete list of all significant DEGs (LFC > 2, padj < 0.05) for both early- and advanced-stage comparisons, including log2 fold change and adjusted p-values, is provided in **Supplementary Table 3** and **Supplementary Table 4**.

### 3.3 Enrichment analysis reveals a stage-specific activation of key signaling networks

**Figure 3:**
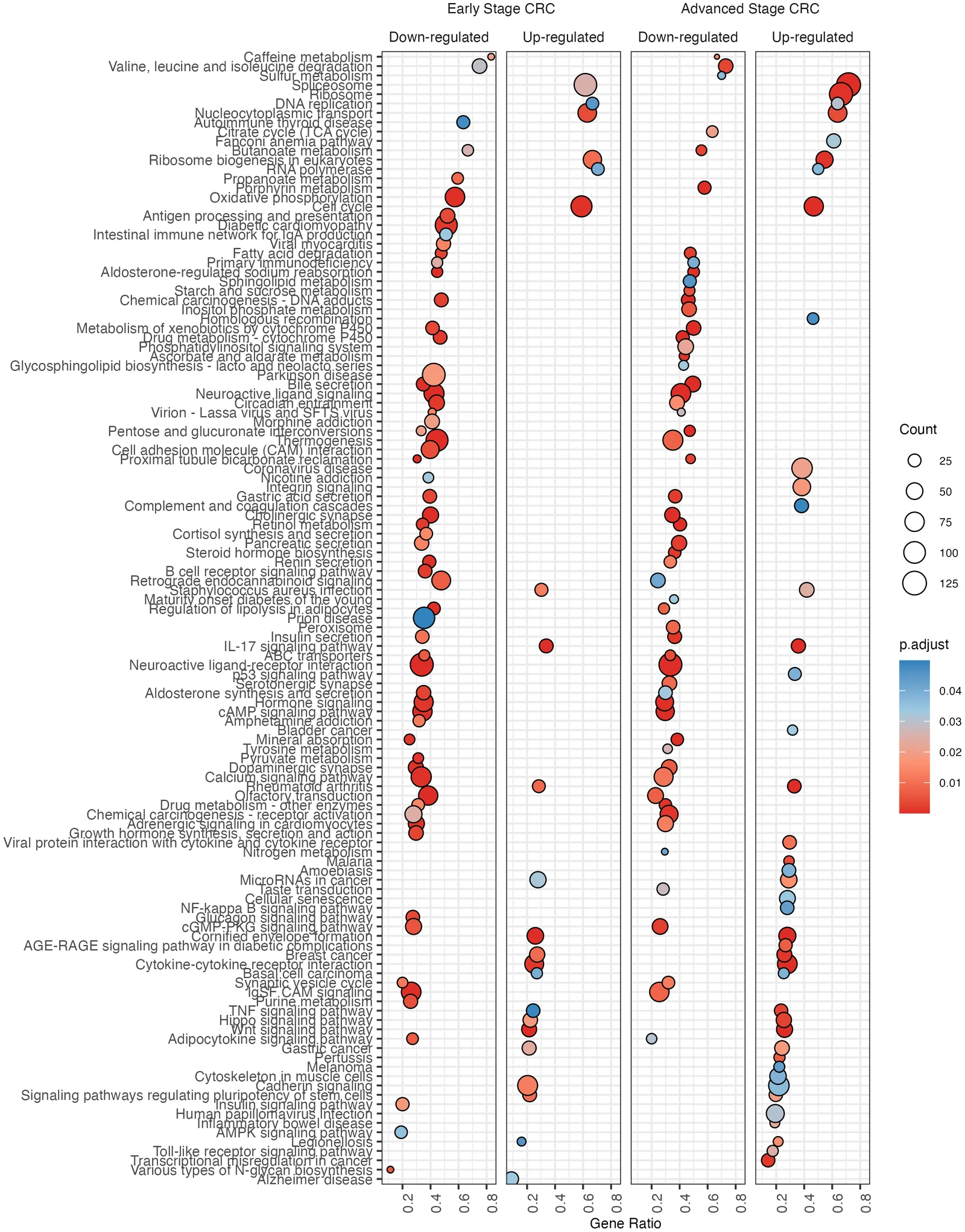
Differential pathway activation during early and advanced stages of colorectal cancer. Gene Set Enrichment Analysis (GSEA) using KEGG database was performed to understand differences in biological pathways on early-stages and advanced-stages CRC compared to adjacent mucosal tissue. Bubble plots illustrate pathway enrichment, with adjusted p-values indicating statistical significance (p < 0.05), gene counts reflecting the number of genes mapped to the pathway, and the gene ratio representing the proportion of genes enriched within each pathway relative to the total genes in that pathway. Pathways were classified into up-regulated or down-regulated based on the normalized enrichment score, reflecting progression-associated molecular alterations.

KEGG pathway enrichment analysis of tumors and adjacent mucosal tissue revealed unique and shared, up and downregulated canonical pathways in early- and advanced-stage (**Figure 3**) CRC. In early-stage CRC, dysregulated pathways included genes related to immune system activation, cellular stress responses, and processes regulating cell growth and development. Upregulated pathways comprise cytokine–cytokine receptor interaction, IL-17 signaling, Tumor Necrosis Factor (TNF) signaling, and Wnt signaling. Downregulated pathways included cytochrome p450-related pathways, purine metabolism, and pyruvate metabolism. In advanced-stage CRC, most immune and oncogenic pathways that were enriched in early-stage remained dysregulated while additional relevant pathways were upregulated: p53 Signaling pathway, cellular senescence, and NF-kappa β signaling.

### 3.4 Gene interaction networks from DEGs

**Figure 4:**
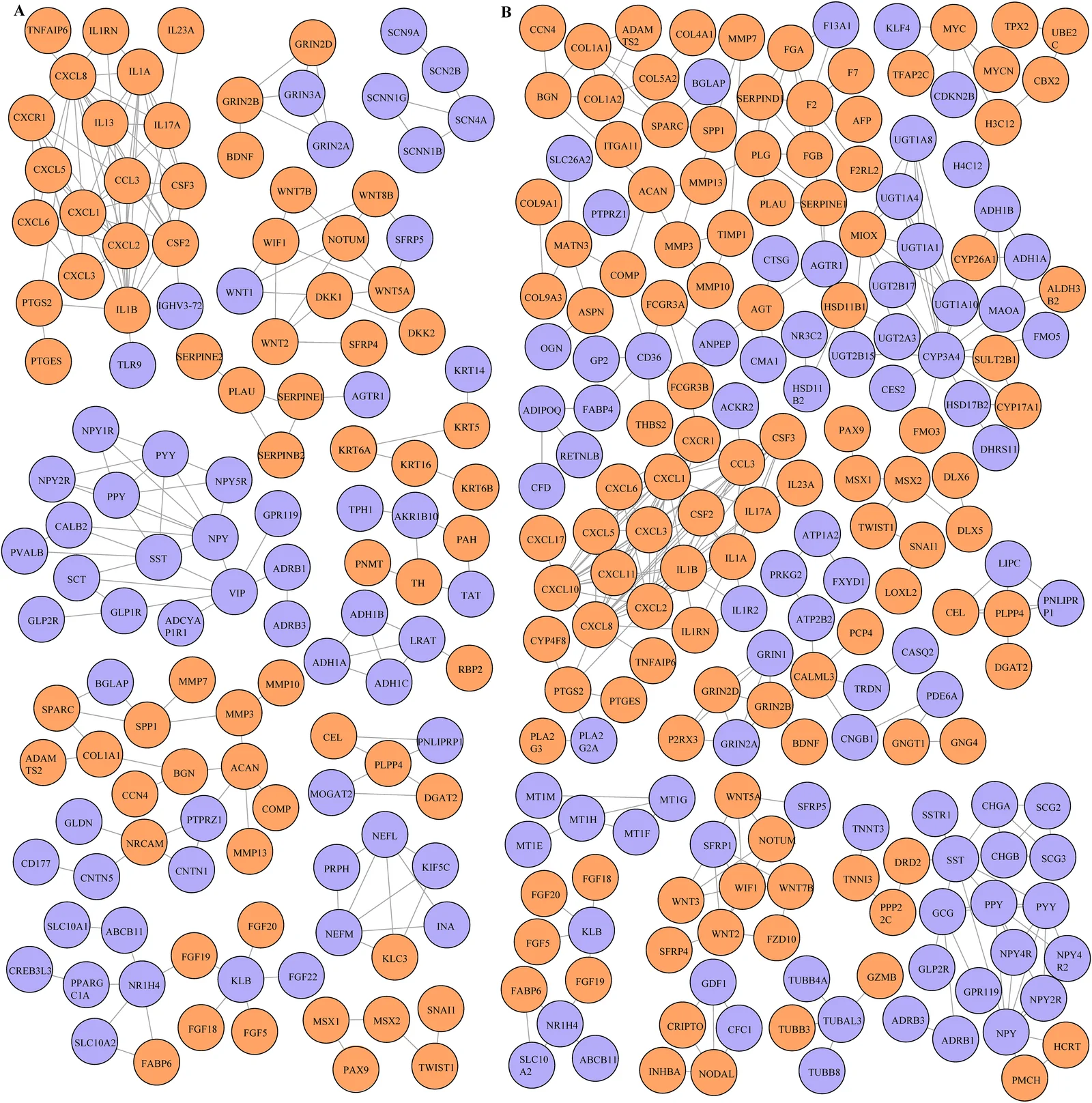
Protein-protein interaction network for DEGs in colorectal cancer. **(A)** Early-stage CRC **(B)** Advanced-stage CRC. DEGs were used to identify functional gene networks using STRINGdb analysis. Networks with less than 5 edges were discarded. The nodes represent proteins inferred from differentially expressed genes, and their color reflects upregulated (orange) or downregulated (blue) genes.

To assess functional relevance between genes based on gene expression data, a network analysis was applied to the transcriptomic data. We used DEGs to construct networks, focusing on connections involving five or more gene interactions. Protein-protein interaction (PPI) network revealed 14 major clusters, with 54.7% proteins that derived from upregulated and 45.3% from downregulated genes in early-stage CRC (**Figure 4**). The analysis of early-stage CRC (**Figure 4A**) showed two major dysregulated subnetworks that included (1) *IL-1A*, *IL-1B*, *IL-17A*, and *CXCL8* and (2) *WNT1*, *WNT2*, *WNT5A*, *DKK1*, and *DKK2* which are genes consistent with activation of innate immune responses. Other dysregulated networks are associated with immune cell recruitment, epithelial barrier integrity, reactive oxygen species production, and pro-inflammatory cytokine signaling pathways. The PPI network analysis of advanced-stage CRC (**Figure 4B**) also revealed 7 major clusters, with 57.6% proteins derived from upregulated and 42.4% from downregulated genes in advanced-stage CRC. The network analysis showed dysregulation of networks related to Wnt/β-catenin signaling, with upregulation of *MYC*, *WNT2*, *WNT3*, and *NOTUM*. Other networks associated with angiogenesis, extracellular matrix degradation, and TGF-driven immunosuppression were dysregulated, showing upregulation of *MMP3*, *MMP7*, *TGFB1*, *VEGFA*, and *SERPINE1*.

### 3.5 Stage-specific expression profiling of wnt signaling genes in an independent cohort

**Figure 5:**
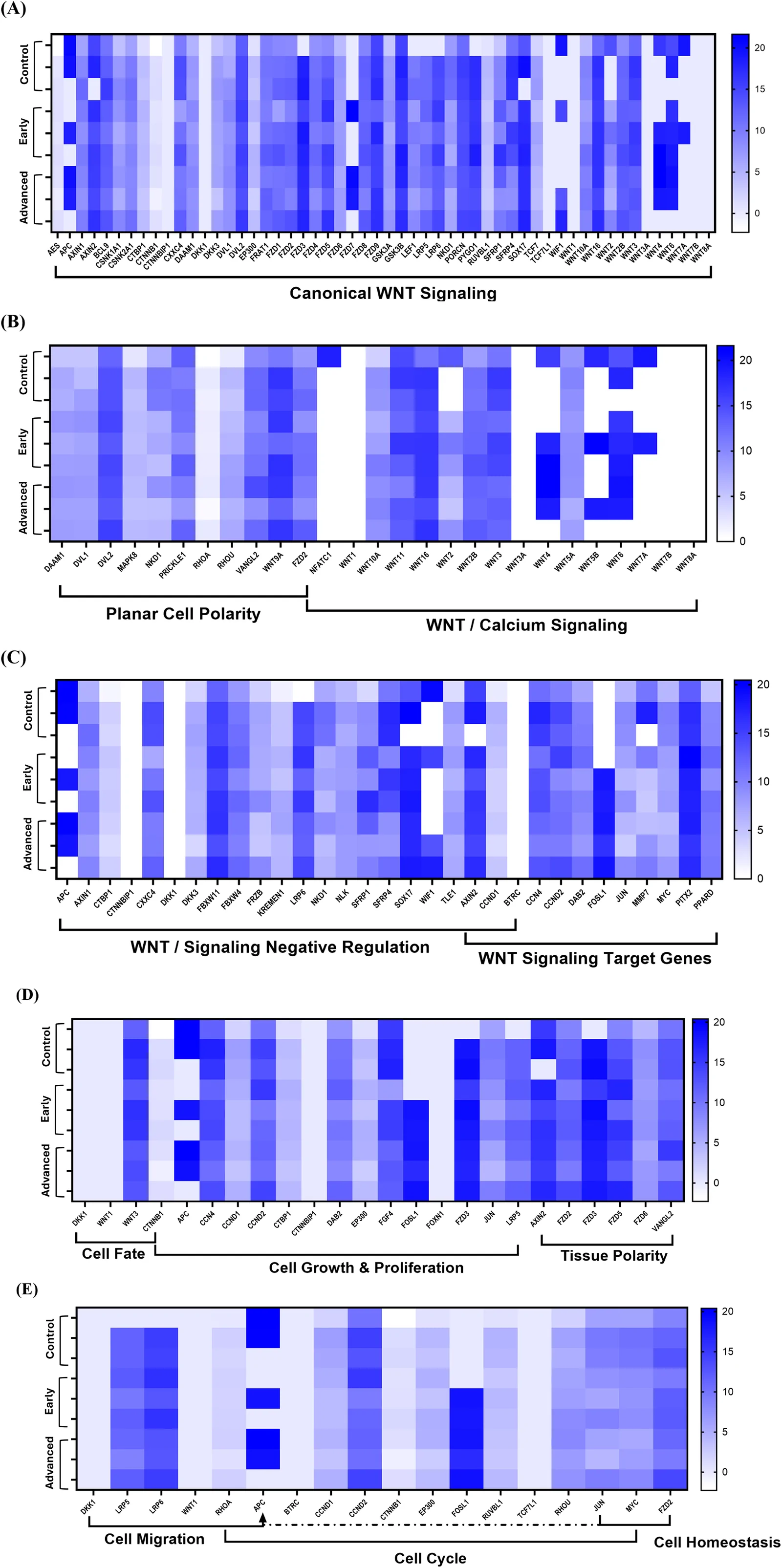
Stage-specific expression profiling of Wnt pathway genes in colorectal cancer reveals progressive dysregulation across canonical and non-canonical branches. Heatmap summary of expression changes in 84 Wnt signaling-related genes across healthy controls, early-stage, and advanced-stage CRC tissues analyzed by LNA-based QuantiNova qPCR. Genes are grouped by functional roles within **(A)** canonical Wnt signaling, **(B)** non-canonical branches (PCP and calcium), **(C)** negative regulators, **(D)** pathways linked to cell fate, proliferation and polarity, and **(E)** cell migration and cell cycle. Expression patterns highlight stage-dependent dysregulation and functional convergence of Wnt signaling in CRC progression. Dashed lines represent shared genes among multiple pathways.

Wnt pathway components identified by RNA-seq were profiled by qPCR in a separate, independent cohort to explore whether comparable expression trends were observed. The expression of 84 Wnt pathway-associated genes was profiled across control, early-stage, and advanced-stage CRC tissues. Transcriptional alterations in the Wnt signaling pathway reveal stage-specific expression patterns, including the dysregulation of genes encoding signaling components, downstream effectors, and regulatory modulators of both the canonical and non-canonical Wnt branches (**Figure 5**). Canonical Wnt signaling components (*WNT4*, *WNT6*, *DVL2*, and *SOX*), alongside transcriptional effectors *PYGO1* and *FZD9* showed higher expression levels in both CRC stages compared to controls (**Figure 5A**). Planar cell polarity pathway genes (*VANGL2*, *PRICKLE1*, *WNT9A*, *DVL2*) and calcium signaling (*WNT4*) also showed higher expression levels in both CRC stages compared to controls (**Figure 5B**). Negative regulators (*SFRP1*, *SOX17*, *APC*, *DKK3*, *CXXC4*, *NKD1*) and signaling targets (*FOSL1*) exhibited higher expression in CRC (**Figure 5C**). Growth and proliferation-associated genes (*CCN4*, *FOSL*, *WNT3*, *CCND2*) and tissue polarity were deregulated (**Figure 5D**). Genes linked to cell cycle regulation, migration, and homeostasis (*APC*, *FOSL1*, *LRP5*, *LRP6*) showed increased expression in early and advanced CRC (**Figure 5E**).

**Figure 6:**
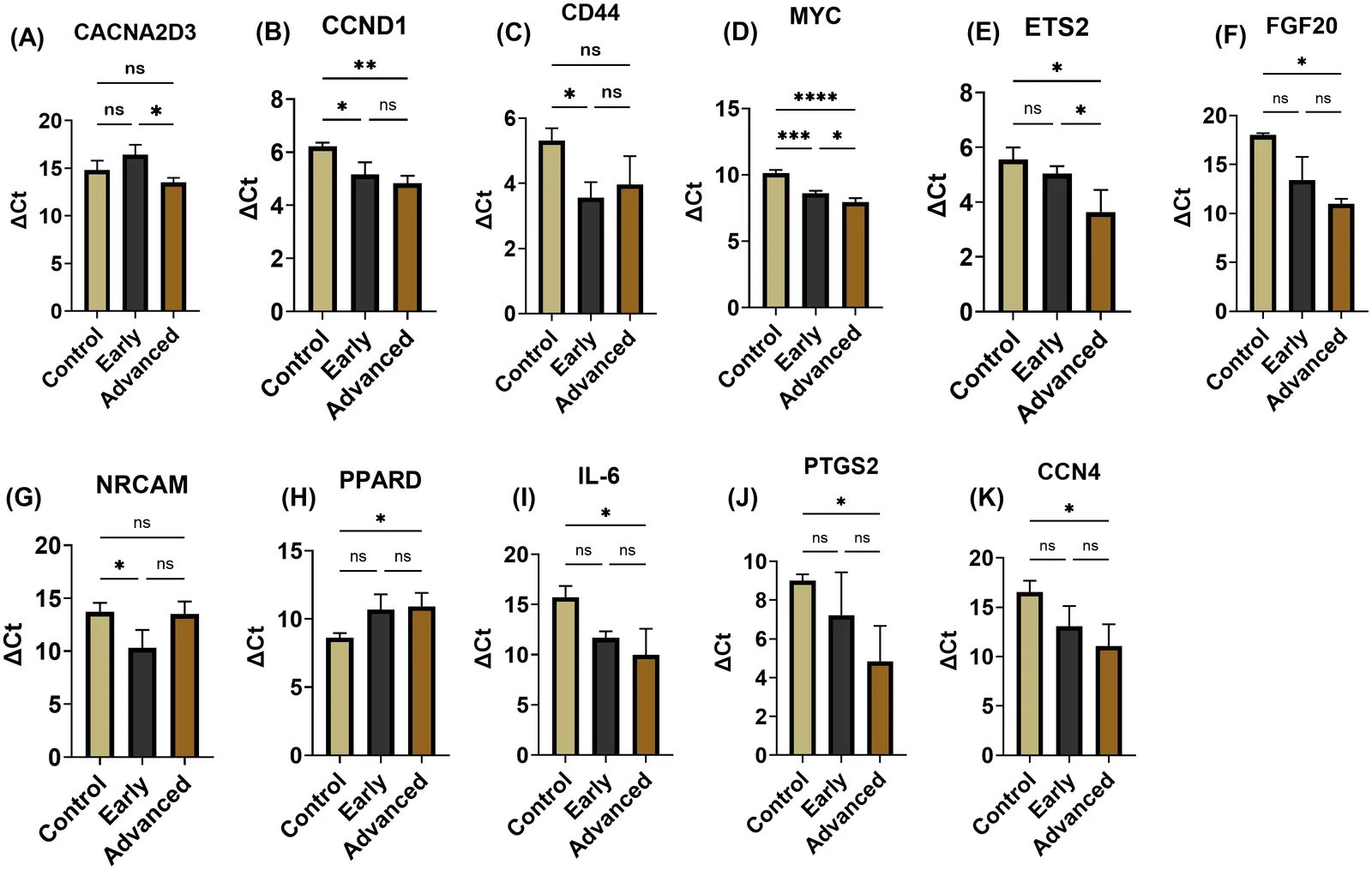
Differential expression of Wnt signaling target genes in early and advanced colorectal cancer analyzed by LNA-based QuantiNova qPCR array. Several Wnt target genes exhibit stage-specific expression patterns, indicating dynamic regulation of the pathway during CRC progression. **(A)** *CACNA2D3*, **(B)** *CCND1*, **(C)** *CD44*, **(D)** *MYC*, **(E)** *ETS2*, **(F)** *FGF20*, **(G)** *NRCAM*, **(H)** *PPARD*, **(I)** *IL6*, **(J)** *PTGS2*, and **(K)** *CCN4*. Significant expression levels were normalized to housekeeping genes and presented as mean ± standard error of the mean (SEM). Low ΔCt values are indicative of high gene expression levels. Statistical significance was determined using one-way ANOVA followed by Tukey’s multiple comparisons test. Asterisks indicate significant differences in expression level (*p < 0.05, ** p < 0.01, ***p < 0.001, and ****p < 0.00001).

**Supplementary Figure 3** and **Figure 6** highlight the qPCRs of Wnt target genes. Among cell cycle-regulating genes, *CCND1* and *MYC* significantly increased in expression with CRC progression (p<0.05) (**Figures 6B, D**). Genes *CD44* and *NRCAM*, which are associated with cell adhesion and tissue remodeling, also significantly increased in expression in early but not in advanced-stage CRC compared to controls (**Figure 6C**; p=0.025 and **Figure 6G**; p=0.040, respectively). In contrast, *CACNA2D3*, a gene encoding a voltage-gated calcium channel subunit, decreased early but increased in advanced-stage CRC compared to controls (**Figure 6A**; p=0.017), indicating stage-specific modulation.

Expression of additional transcriptional and inflammatory mediators was measured. Increased expression of the transcription factor *ETS2*, as well as genes implicated in epithelial differentiation and immune modulation (*FGF20* and *IL6*) were observed in advanced-stage CRC compared to controls (**Figures 6E, F, I**; p<0.05). *PTGS2* (*COX-2*), which is regulated by both Wnt and NF-κB signaling, was significantly upregulated in advanced-stage CRC compared to controls (**Figure 6J**; p=0.049). In contrast to the overall trend of Wnt target gene upregulation, *PPARD* (**Figure 6H**; p=0.042), a nuclear receptor involved in metabolism, differentiation, and Wnt-mediated transcriptional regulation, was significantly downregulated in advanced CRC. Finally, *CCN4* (**Figure 6K**, *WISP1*; p=0.026), a matricellular protein and direct Wnt target, was also significantly upregulated in advanced CRC stage.

## 4 Discussion

Immunological and molecular mechanisms are central to CRC progression by influencing tumor-host interactions that dynamically change throughout CRC development. In this study, transcriptomic profiling of CRC from PRH revealed stage-specific gene expression signatures, that is consistent with a transition from an immune-active, pro-inflammatory tumor microenvironment in early-stage CRC to an immune-suppressive, pro-metastatic state in advanced-stage (stage III) disease. This transition is characterized by a progressive dysregulation of both canonical and non-canonical Wnt signaling components, with concurrent activation of oncogenic effectors and compensatory antagonists in early disease shifting toward fixed β-catenin-driven transcriptional programs in advanced stages (45). Our findings highlight the relevance of stage-specific expression patterns in CRC and a population-specific framework for stage-adapted biomarker discovery candidates and therapeutic stratification.

Our results suggest that early-stage CRC in the PRH cohort is marked by expression of inflammatory mediators such as *IL1A*, *IL1B*, and *CXCL8* (IL-8), consistent with active immune responses and epithelial barrier disruption. Pathway enrichment and network analyses is consistent with an immune-active profile resembling the immune-mediated tumor-promoting inflammation described by Grivennikov et al. (2010), where the initial cytokine-mediated responses did not appear to inhibit tumor growth but instead promoted tumor progression through mechanisms involving chronic inflammation, or immunoediting (46). The simultaneous upregulation of matrix remodeling enzymes such as MMP-3, in early-stage tumors supports this model, as early extracellular matrix remodeling may facilitate immune cell infiltration while also priming the tissue for subsequent invasion (47). Together, these observations correlate with the dual role of inflammation in CRC, protective in early disease but progressively hijacked to support tumor growth and immune evasion.

While our findings identify distinct signatures within these cohorts, it should be noted that advanced stage samples within the study were exclusively Stage III. Conclusions regarding advanced stage signatures should be interpreted within the context of this specific clinical context. Advanced stage tumors showed transcriptional enrichment for *TGFB1*, *VEGFA*, *SERPINE1*, and genes involved in leukocyte transendothelial migration, chemokine signaling, and tight junction remodeling. These changes may reflect a TME characterized by angiogenesis, epithelial-mesenchymal transition, and impaired anti-tumor activity. STRING network analysis further revealed activation markers of TGF-β, Wnt/β-catenin, and immune regulatory checkpoint pathways, consistent with well-established mechanisms of metastatic progression and immune escape (20). Notably, advanced-stage CRC retained inflammatory signaling, but its specific functional role in tumor progression in PRH remains to be fully elucidated. Increased expression of *IL6* and *PTGS2* (COX2), alongside immunosuppressive mediators like TGF-β1, suggests that chronic inflammation persists but is repurposed to promote immune tolerance rather than tumor control. These stage-specific transitions suggest hypotheses for stage-adapted therapy that would require functional and clinical validation. Given the retained immune activity of early-stage tumors, immune-stimulating approaches (IL-15 or TLR agonists) could be worth investigating, whereas the immunosuppressive profile of advanced-stage disease suggests combination strategies such as PD-1 blockade with TGF-β inhibitors may merit exploration (48). Importantly, this immune remodeling does not occur in isolation but intersects with fundamental tumor-signaling pathways that shape colorectal cancer progression, particularly Wnt signaling. Similar patterns in expression profiles and pathways alterations in CRC have been reported in previous RNA seq studies (49–51). The unique molecular profile of the PRH population with CRC exhibits a significantly higher mutational burden in genes like *APC, TP53*, and *KRAS* (14), which has been associated with constitutive activation of the Wnt pathway and CRC progression (52–54) In the PRH population, *APC* truncating mutations have been reported at higher frequency than other cohorts, a pattern consistent with constitutive Wnt/β-catenin activation as described in the literature (52, 53). Elevated *KRAS* mutation frequency has been associated with MAPK pathway dysregulation and increased tumor progression (52), and TP53 mutations have been linked to impaired checkpoint control that may allow co-occurring oncogenic alterations to accumulate. While direct causal inference from our transcriptomic data is not possible, the expression patterns observed are consistent with these mutation-associated pathway consequences.

**Figure 7:**
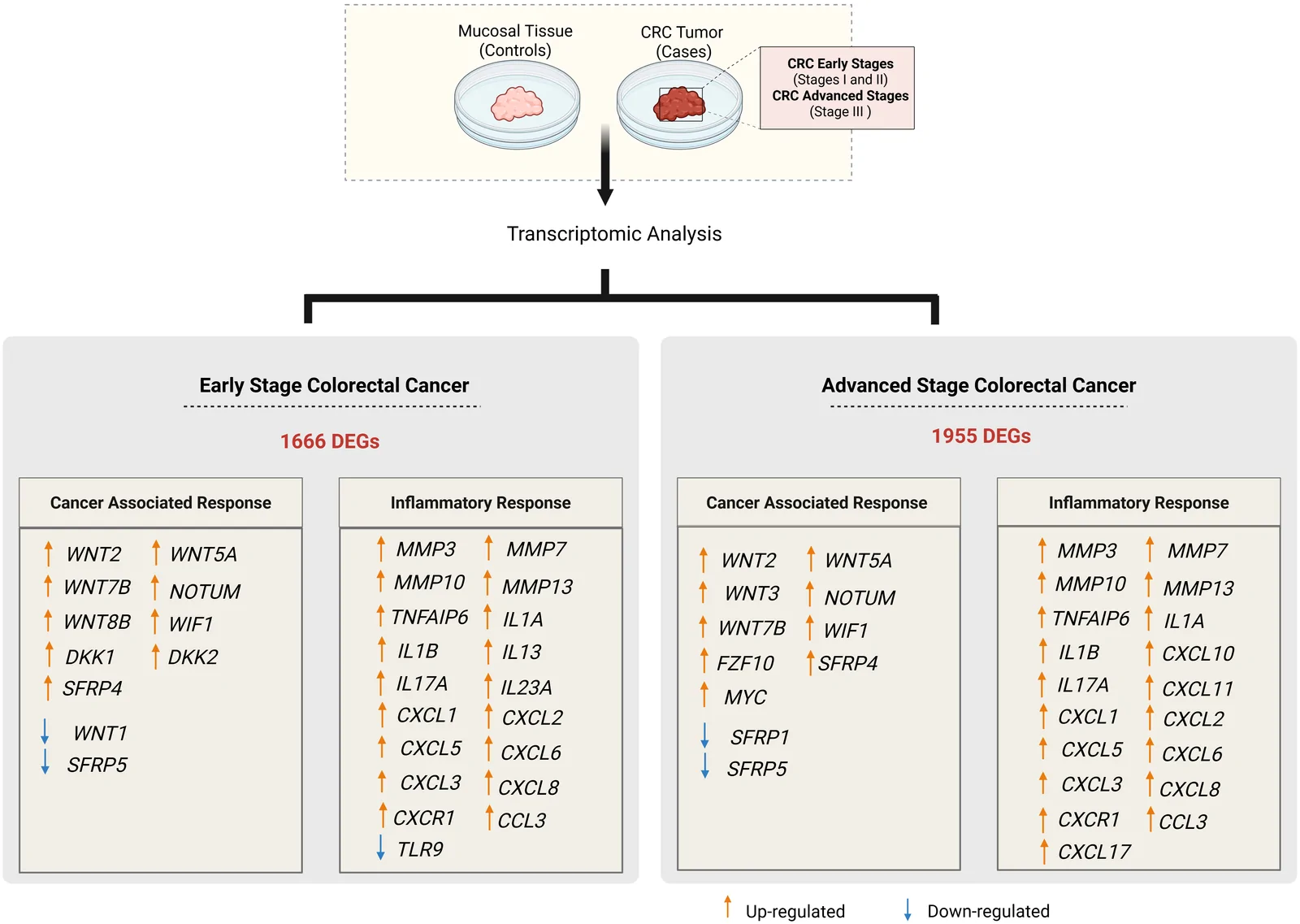
Stage-specific transcriptomic signatures reveal progressive activation of tumor-associated and inflammatory pathways in CRC. Transcriptomic analysis of colorectal cancer tissues reveals stage-specific gene expression patterns. Early-stage CRC tumors show activation of oncogenic and inflammatory genes, while advanced stage (stage III) exhibit broader immune dysregulation and chemokine signaling, reflecting tumor progression and microenvironment remodeling. Upregulated and downregulated genes are indicated with orange and blue arrows, respectively. Created in BioRender. Zenón, C. (2026) https://BioRender.com/ccn8b7d. Publication licensed under CC BY 4.0.

The Wnt pathway is described as a highly conserved signaling that plays a key role in the pathogenesis, development, and progression of colorectal cancer (55). Numerous studies have shown the involvement of canonical and non-canonical pathway regulation in CRC and the relevance in cancer progression according to the ligand and pathway fate (56). Dysregulation of the Wnt signaling components promotes changes in MMPs expression downstream in the Wnt pathway, impacting biological processes such as tissue remodeling (57). Interestingly, transcriptomic changes observed in our data highlight the dynamic changes of Wnt signaling in CRC progression. Differential gene expression analysis revealed stage-specific upregulation of both canonical and non-canonical Wnt pathway components in early and advanced stages of disease (**Figure 7**). Notably, transcriptional alterations in the early stages included upregulation of *WNT2*, *WNT8B*, and *MYC*, consistent with activation of the canonical Wnt/β-catenin pathway, which is well-established in promoting proliferation and tumor progression. Simultaneously, genes associated with the non-canonical pathway (*WNT5A* and *WNT7B*) were differentially expressed, indicating that both arms of the Wnt signaling network are engaged during CRC evolution. Interestingly, early-stage tumors also exhibited increased expression of Wnt antagonists such as *WIF1*, *DKK1*, and *SFRP4*, suggesting a scenario in which oncogenic activation and compensatory inhibition coexist. This simultaneous activation is consistent with early neoplastic regulation of cells trying to counterbalance the tumor progression (18, 58). Additionally, our results reveal upregulation of canonical ligands (*WNT3A*, *WNT10A*), receptors (*FZD7*, *FZD9*), and downstream effectors (*LEF1*, *CCND1*) in advanced CRC. Importantly, we also observed dysregulation of known Wnt antagonists (*SFRP1* and *APC*) and distinct expression patterns in non-canonical components (*WNT5A*, *SFRP4*), suggesting functional rewiring of the Wnt signaling network as CRC advances. The transition from signaling plasticity in early stages to fixed β-catenin–driven transcriptional programs in later stages, outlines previously described in microsatellite-stable CRC tumors (59), and may offer population-specific therapeutic entry points in PRH patients. Remarkably, the Wnt target genes *MYC* and *CCND1*, genes frequently used as monitors of Wnt activity, were upregulated in the most advanced disease states. This is likely to represent a progressive, undifferentiated state (60, 61). Moreover, the downregulation of *PPARD*, a nuclear receptor that is modulated by Wnt signaling and is associated with late-onset tumorigenesis, adds to that trajectory (62). Together, these findings highlight progressive reprogramming of signaling networks rather than a simple increase in pathway activation.

Specifically, our results reveal a dysregulation of WNT3A, WNT10A, MYC, FZD7, FZD9, and DKK1, consistent with constitutive APC loss-of-function; upregulation of CXCL8 and PTGS2 (COX-2) aligns with KRAS driven inflammatory signaling (63); elevated IL1B and IL6 is consistent with TP53 mutation-associated immune dysregulation (64); and increase SERPINE1 expression reflects SMAD4 mediated TGF-β pathway activation (65). Together, these mutation-linked expression patterns suggest a distinct molecular landscape in PRH CRC that may contribute to the stage-specific Wnt pathway activation observed in our data. This pattern contrasts with the transcriptomics findings in other non-European ethnic cohorts, such as a Taiwanese CRC study where reduced Wnt signaling was linked to worse outcomes, suggesting that both the direction and stage-specific role of Wnt dysregulation in CRC can vary across populations (66). Furthermore, the early-stage activation of pro-inflammatory cytokines (*IL-1A, IL-1B, CXCL8*) alongside Wnt antagonists (*WIF1*, *DKK1*, *SFRP4*) observed in our PRH cohort has not been reported in predominantly non-Hispanic transcriptomic studies, pointing to a population specific dynamic in the crosstalk between inflammatory signaling and Wnt pathway regulation.

While this study provides the first dedicated stage-stratified transcriptomic characterization of CRC in the PRH population, several limitations must be acknowledged. First, the small cohort size (n=15 discovery cohort, n=9 independent cohort) limits statistical power for subgroup analyses; formal re-stratification by TNM components, anatomical subsite, or advanced substage was not statistically feasible given that the advanced-stage transcriptomics cohort comprised exclusively Stage III patients (n=8), with no Stage IV cases enrolled at the time of analysis, and conclusions regarding advanced-stage signatures should be interpreted within this specific clinical context. Second, the qPCR confirmation cohort used independent healthy control tissues rather than matched adjacent mucosa from the same patients, as RNA was exhausted following library preparation for the discovery cohort; the qPCR data are therefore presented as an independent, exploratory expression survey in a separate cohort rather than technical validation of the RNA-seq differential expression results. Anatomical subsite was also unevenly distributed across stages in the independent cohort: early-stage tumors were exclusively colonic, whereas advanced-stage tumors included rectal and rectosigmoid sites, and the subsite of the healthy control tissues was not documented at the time of PURIFICAR procurement. Because the right colon, left colon, and rectum differ substantially in transcriptomic and immune biology, reflecting differences in embryological origin, microbiota, and molecular-subtype distribution (67, 68); tumor stage and subsite are partially confounded in this cohort. Consequently, some of the expression differences attributed here to disease stage may also reflect, in part, differences in tumor location rather than stage alone. We therefore interpret the stage-specific expression patterns from the independent cohort as preliminary, and we identify subsite-matched sampling and systematic documentation of control-tissue origin as priorities for future work. Third, PCA confirmed robust global separation between tumor and adjacent tissue compartments across all samples, underscoring the transcriptomic distinctiveness of each compartment. The potential influence of field cancerization, whereby molecular alterations have been documented in histologically normal mucosa proximal to the tumor margin cannot be fully excluded; however, this reflects an inherent biological feature of colorectal carcinogenesis rather than a compromise in tissue classification (69, 70). Fourth, survival data was not systematically captured in PURIFICAR at the time of sample procurement, precluding correlation of transcriptomic signatures with clinical outcomes; this is identified as a priority for future prospective work. Fifth, this study relies exclusively on RNA-level data; protein-level validation and functional characterization of key candidates, including MAGEA4, WNT2, CCND1, and IL6, are identified as priorities for follow-up studies with expanded cohorts currently being recruited through PURIFICAR. Despite these limitations, the present work offers a preliminary, hypothesis-generating transcriptomic framework to guide future mechanistic and translational research in the PRH population.

Beyond molecular findings, our results reveal a distinct biological landscape in PRH CRC, including unique transcriptomic signatures, immune cytokine alterations, and stage-specific gene expression patterns that diverge from profiles reported in non-Hispanics cohorts, providing direct molecular evidence of biological disparities in this population (21). Prior molecular profiling studies have shown that differences in the prevalence of molecular markers among PRH tumors compared to other racial and ethnic groups suggest a distinct carcinogenic pathway in this population (14), yet the transcriptomic and immunological dimensions of this had not been characterized. These findings reinforce the urgent need to address the underrepresentation of PRHs in genomic databases and clinical trials, particularly given the disproportionately high CRC mortality this population experiences relative to other Latino groups in the United States and Latin America (17, 71). Integrating molecular profiling with social determinants of health will be essential to develop equitable models of cancer prevention, diagnosis, and treatment. Future studies will focus on expanding the sample size, incorporating a multi-omics integration, employing quantitative proteomic approaches, and functional validations in larger cohorts, steps that will be critical to further define the regulatory mechanisms driving CRC progression in the PRH population and establish a foundation for personalized therapeutic strategies.

In conclusion, this study identifies stage-specific immune and Wnt signaling alterations in PRH CRC, revealing a transition from immune activation in early-stage disease to immune suppression in advanced-stage (stage III) disease accompanied by progressive rewiring of oncogenic signaling networks. Because the advanced stage cohort comprised exclusively stage III cases, these findings are stage III-specific; their extension to metastatic (stage IV) disease, which was not sampled, remains to be determined. Molecular changes in the Wnt pathway differ between Hispanic/Latino and non-Hispanic white CRC patients in ways that may influence how cancer develops and responds to treatment and highlighted the insufficient molecular profiling of Myc and Wnt signaling in this group (72, 73). However, the stage-specific transcriptomic patterning of these pathways in the PRH population specifically remains uncharacterized; a gap this study begins to address. These findings provide a population-specific framework for biomarker discovery and therapeutic stratification and highlight the importance of pairing molecular precision with equitable systems that ensure fair access and representation. The future of precision medicine must extend beyond the genome and not only recognize the inequities within the system, but more broadly address the socio-economic inequities that drive them (74).

## Data Availability

The datasets presented in this study can be found in online repositories. The names of the repository and accession number can be found below: https://www.ncbi.nlm.nih.gov/geo/query/acc.cgi?acc=GSE325868, GSE325868.

## Supplementary material

The Supplementary Material for this article can be found online at: https://www.frontiersin.org/articles/10.3389/fimmu.2026.1838210/full#supplementary-material

Supplementary Figure 1 Principal Component Analysis (PCA) of transcriptomic profiles. PCA was performed to evaluate and visualize the distribution of samples based on global gene expression patterns. Each point represents an individual sample, plotted according to the first two principal components, which capture the largest sources of variation in the transcriptomic dataset. Clustering samples reflects similarities in overall gene expression profiles **(A)** in early CRC stage **(B)** and advanced **(C)**.

Supplementary Figure 2 Top 10 Differentially expressed genes in early and advanced CRC stages. Bar plots depict the top 10 upregulated (orange) genes and downregulated (blue) genes identified in the Transcriptomic analysis. Data is represented as log2 fold change.

Supplementary Figure 3 Stage-Specific Expression Profiling of Wnt Target Genes in Colorectal Cancer. This heatmap illustrates the expression profiles of canonical Wnt signaling target genes across different experimental groups. Rows represent individual Wnt target genes, and columns correspond to biological samples or treatment conditions. Gene expression values are presented as normalized and log-transformed expression levels, with a color gradient indicating relative expression (blue: high; white: low). This analysis highlights differential regulation of Wnt targets associated with **(A, C)** differentiation and development (growth factors, cell cycle, cell adhesion molecules, and cell migration); **(D)** signal transduction (Wnt signaling and TGF-β signaling), and **(E)** transcription factors.
