# Supplementary material for: Integrated transcriptomic profiling reveals immune and molecular stage-specific signatures of colorectal cancer in hispanics living in Puerto Rico
Source: Front Immunol. 2026 Aug 11;17:1838210. doi: 10.3389/fimmu.2026.1838210 (PMC13503336; doi:10.3389/fimmu.2026.1838210)
Supplement: Supplementary file 1 [file Table1.docx]

**Supplementary Table 1: Characteristics of RNA participants (Discovery Cohort)**

| **Characteristic** | **N = 15***^1^* |
| --- | --- |
| **Stage** |  |
| Advanced | 8.0 (53.3%) |
| Early | 7.0 (46.7%) |
| **Age at Recruitment** | 63.5 (41.1, 84.5) |
| **Age at CRC Diagnosis** | 61.0 (41.0, 84.0) |
| **Age at Tissue Collection** | 63.0 (41.0, 84.0) |
| **Sex** |  |
| Male | 9.0 (60.0%) |
| Female | 6.0 (40.0%) |
| **Health Insurance (N=12)** |  |
| Medicare | 1.0 (8.3%) |
| Medicare Advantage | 2.0 (16.7%) |
| Private | 9.0 (75.0%) |
| **Education (N=10)** |  |
| Less than Bachelors Degree | 4.0 (40.0%) |
| Bachelors Degree or Higher | 6.0 (60.0%) |
| **Marital status (N=10)** |  |
| Single/Divorced/Widowed | 3.0 (30.0%) |
| Married or Living Together | 7.0 (70.0%) |
| **Weight (lbs) (N=14)** | 153.5 (105.0, 270.0) |
| **Height (in) (N=13)** | 65.0 (57.0, 80.0) |
| **BMI (N=13)** | 25.8 (17.5, 34.0) |
| **Diabetes (N=14)** | 4.0 (28.6%) |
| **Aspirin 3 times per week (N=14)** | 2.0 (14.3%) |
| **NSAIDs 3 times per week (N=14)** | 2.0 (14.3%) |
| **Statins 3 times per week (N=10)** | 3.0 (30.0%) |
| **Lifetime Smoker (>100 cigarettes) (N=14)** | 5.0 (35.7%) |
| **Lifetime Alcohol Consumption (>12 drinks) (N=13)** | 8.0 (61.5%) |
| **Family Member Diagnosed with Any Cancer (Except Non-Melanoma Cancer) (N=14)** | 8.0 (57.1%) |
| **Family History of Lynch Syndrome-Associated Cancers (N=14)** | 1.0 (7.1%) |
| **Family Member Diagnosed with Colorectal Cancer (N=14)** | 1.0 (7.1%) |
| **Colorectal Surgery Pathology** | 15.0 (100.0%) |
| **Colorectal Surgery Date (Year)** | 2016 (2011, 2019) |
| **Surgery Location** |  |
| Colon |  |
| Left | 10.0 (66.7%) |
| Right | 2.0 (13.3%) |
| Unspecified | 2.0 (13.3%) |
| Rectum | 1.0 (6.7%) |
| **Tumor grade** |  |
| Low | 14.0 (93.3%) |
| Undetermined | 1.0 (6.7%) |
| **Colorectal Tumor T** |  |
| T1 | 2.0 (13.3%) |
| T2 | 2.0 (13.3%) |
| T3 | 10.0 (66.7%) |
| T4b | 1.0 (6.7%) |
| **Colorectal Tumor N** |  |
| N0 | 6.0 (40.0%) |
| N1 | 2.0 (13.3%) |
| N1a | 3.0 (20.0%) |
| N1b | 3.0 (20.0%) |
| N2b | 1.0 (6.7%) |
| **Colorectal Tumor M** |  |
| M0 | 15.0 (100.0% |
| **Tumor Stage** |  |
| Stage I | 3.0 (20.0%) |
| Stage IIA | 2.0 (13.3%) |
| Stage IIB | 2.0 (13.3%) |
| Stage III | 1.0 (6.7%) |
| Stage IIIA | 1.0 (6.7%) |
| Stage IIIB | 5.0 (33.3%) |
| Stage IIIC | 1.0 (6.7%) |
| *^1^*n (%); Median (Min, Max) | |
