# Supplementary material for: Integrated transcriptomic profiling reveals immune and molecular stage-specific signatures of colorectal cancer in hispanics living in Puerto Rico
Source: Front Immunol. 2026 Aug 11;17:1838210. doi: 10.3389/fimmu.2026.1838210 (PMC13503336; doi:10.3389/fimmu.2026.1838210)
Supplement: Supplementary file 2 [file Table2.docx]

**Supplementary Table 2: Characteristics of PCR participants (Independent Cohort)**

| **Characteristic** | **Control**  N = 3*^1^* | **Early**  N = 3*^1^* | **Advanced**  N = 3*^1^* | **p-value***^2^* |
| --- | --- | --- | --- | --- |
| **Age at Recruitment** | - | 61.9 (56.9, 66.9) | 65.2 (61.6, 68.8) | 0.4 |
| Unknown | 3 | 0 | 0 |  |
| **Age at CRC Diagnosis** | - | 61.5 (57.0, 66.0) | 61.5 (55.0, 68.0) | >0.9 |
| **Age at tissue sampling** | 57.0 (52.0, 58.0) | 66.0 (56.0, 73.0) | 61.0 (53.0, 68.0) | 0.4 |
| **Sex** |  |  |  | >0.9 |
| Male | 2.0 (66.7%) | 2.0 (66.7%) | 3.0 (100.0%) |  |
| Female | 1.0 (33.3%) | 1.0 (33.3%) | 0.0 (0%) |  |
| **Health Insurance** |  |  |  | 0.2 |
| Private | 1.0 (33.3%) | 2.0 (100.0%) | 2.0 (100.0%) |  |
| Reforma | 2.0 (66.7%) | 0.0 (0.0%) | 0.0 (0.0%) |  |
| Unknown | 0 | 1 | 1 |  |
| **Education** |  |  |  | >0.9 |
| Less than Bachelors Degree | 1.0 (33.3%) | 1.0 (100.0%) | 0.0 (0.0%) |  |
| Bachelors Degree or Higher | 2.0 (66.7%) | 0.0 (0.0%) | 1.0 (100.0%) |  |
| Unknown | 0 | 2 | 2 |  |
| **Weight (lbs)** | 160.0 (150.0, 178.0) | 138.0 (135.0, 218.0) | 203.0 (200.0, 203.0) | 0.3 |
| **Height (in)** | 65.0 (64.0, 69.0) | 68.0 (60.0, 69.0) | 69.0 (66.0, 71.0) | 0.4 |
| **BMI** | 25.7 (23.6, 29.6) | 26.4 (21.0, 32.2) | 29.5 (28.3, 32.8) | 0.4 |
| **Diabetes** | 0.0 (0.0%) | 1.0 (50.0%) | 0.0 (0.0%) | 0.6 |
| Unknown | 0 | 1 | 1 |  |
| **Aspirin 3 times per week** |  |  |  |  |
| No | 2.0 (100.0%) | 3.0 (100.0%) | 3.0 (100.0%) |  |
| Unknown | 1 | 0 | 0 |  |
| **NSAIDs 3 times per week** | 0.0 (0.0%) | 2.0 (66.7%) | 0.0 (0.0%) | 0.3 |
| **Statins 3 times per week** | 0.0 (0.0%) | 1.0 (50.0%) | 0.0 (0.0%) | >0.9 |
| Unknown | 1 | 1 | 1 |  |
| **Lifetime Smoker (>100 cigarettes)** | 1.0 (33.3%) | 2.0 (66.7%) | 2.0 (66.7%) | >0.9 |
| **Lifetime Alcohol Consumption (>12 drinks)** | 2.0 (66.7%) | 1.0 (50.0%) | 2.0 (66.7%) | >0.9 |
| Unknown | 0 | 1 | 0 |  |
| **Family Member Diagnosed with Colorectal Cancer** | 1.0 (50.0%) | 1.0 (33.3%) | 2.0 (66.7%) | >0.9 |
| Unknown | 1 | 0 | 0 |  |
| **Colorectal Surgery Date (Year)** | 2019 (2014, 2020) | 2014 (2009, 2016) | 2014 (2008, 2014) | 0.2 |
| **Surgery Location** |  |  |  | 0.1 |
| Colon |  |  |  |  |
| Left | - | 1.0 (33.3%) | 3.0 (100.0%) |  |
| Right | - | 0.0 (0.0%) | 0.0 (0.0%) |  |
| Unspecified | - | 1.0 (33.3%) | 0.0 (0.0%) |  |
| Rectum | - | 1.0 (33.3%) | 0.0 (0.0%) |  |
| **Tumor grade** |  |  |  | >0.9 |
| Low | - | 2.0 (66.7%) | 2.0 (66.7%) |  |
| Undetermined | - | 1.0 (33.3%) | 0.0 (0.0%) |  |
| Unspecified | - | 0.0 (0.0%) | 1.0 (33.3%) |  |
| **Colorectal Tumor T** |  |  |  | >0.9 |
| T1 | - | 1.0 (33.3%) | 0.0 (0.0%) |  |
| T2 | - | 0.0 (0.0%) | 1.0 (33.3%) |  |
| T3 | - | 2.0 (66.7%) | 2.0 (66.7%) |  |
| **Colorectal Tumor N** |  |  |  | 0.10 |
| N0 | - | 3.0 (100.0%) | 0.0 (0.0%) |  |
| N1 |  | 0.0 (0.0%) | 1.0 (33.3%) |  |
| N1b |  | 0.0 (0.0%) | 2.0 (66.7%) |  |
| **Colorectal Tumor M** |  |  |  |  |
| M0 | - | 3.0 (100.0%) | 3.0 (100.0%) |  |
| **Tumor Stage** |  |  |  | 0.4 |
| Stage I | - | 1.0 (33.3%) | 0.0 (0.0%) |  |
| Stage IIA | - | 2.0 (66.7%) | 0.0 (0.0%) |  |
| Stage III | - | 0.0 (0.0%) | 1.0 (33.3%) |  |
| Stage IIIA | - | 0.0 (0.0%) | 1.0 (33.3%) |  |
| Stage IIIB | - | 0.0 (0.0%) | 1.0 (33.3%) |  |
| *^1^*n (%); Median (Min, Max) | | | | |
| *^2^*Fisher's exact test; Kruskal-Wallis rank sum test | | | | |
